# Molecular detection and characterization of *Anaplasma marginale* in cattle from the Lower Shabelle Region of Somalia

**DOI:** 10.1186/s13071-026-07563-2

**Published:** 2026-07-23

**Authors:** Aamir M. Osman, Ahmed A. Hassan-Kadle, Marcos R. André, Kelly A. Brayton, Abdalla M. Ibrahim, Anna Claudia B. Mongruel, Flávia C. M. Collere, Caroline Tostes Secato, Daniel Antônio Braga Lee, Lorena Freitas das Neves, Renan Bressianini do Amaral, Thállitha S. W. J. Vieira, Rosangela Z. Machado, Rafael F. C. Vieira

**Affiliations:** 1 Graduate Program in Veterinary Sciences, Universidade Federal do Paraná, Curitiba, Paraná Brazil; 2 Somali One Health Centre, Mogadishu, Somalia; 3 Department of Animal Health and Veterinary Services, Ministry of Livestock, Forestry, and Range, Mogadishu, Somalia; 4 Abrar Research and Training Centre, Mogadishu, Somalia; 5 Departamento de Patologia, Faculdade de Ciências Agrárias e Veterinárias, Reprodução e Saúde Única, Universidade Estadual Paulista (FCAV/UNESP), Jaboticabal, Brazil; 6 Program in Vector-borne Disease, Department of Veterinary Microbiology and Pathology, Washington State University, Pullman, WA USA; 7 Department of Veterinary Tropical Diseases, Faculty of Veterinary Science, University of Pretoria, Onderstepoort, South Africa; 8 Department of Veterinary Medicine, Federal University of Paraná, Palotina, PR Brazil; 9 Department of Chemistry, The University of North Carolina at Charlotte, Charlotte, USA; 10 Department of Public Health and Health Administration, The University of North Carolina at Charlotte, Charlotte, USA; 11 Center for Computational Intelligence to Predict Health and Environmental Risks (CIPHER), The University of North Carolina at Charlotte, Charlotte, USA

**Keywords:** Anaplasmosis, *msp1α*, *msp1β*, *msp4*, *Rhipicephalus pulchellus*, Sub-Saharan Africa

## Abstract

**Background:**

*Anaplasma marginale* is a major tick-borne pathogen impacting the global cattle industry, yet its status in Somalia remains poorly understood. The present study aimed to investigate the molecular and serological occurrence and the genetic diversity of *A. marginale* in cattle from the Lower Shabelle region of Somalia.

**Methods:**

Blood samples (*n* = 131) were collected from cattle, alongside epidemiological data on location, body condition, and tick presence. Packed cell volume (PCV) was measured, and sera were screened for anti-*A. marginale* IgG antibodies using a commercial rMSP5-based ELISA. Molecular detection was performed by qPCR targeting the *msp1β* gene, and positive samples were further characterized by PCR amplification and sequencing of the *msp1α* and *msp4* genes, followed by bioinformatic analyses.

**Results:**

A total of 110/131 (83.9%) cattle presented IgG antibodies to *A. marginale*, while 121/131 (92.4%) tested positive by qPCR targeting the *msp1β* gene. A significant difference in median PCV was observed between seropositive and seronegative animals (*p* = 0.026). qPCR positivity showed no significant association with tick infestation and body condition. There was a fair agreement between serology and qPCR (*κ* = 0.28). Among the 32 qPCR-positive samples further analyzed, 31 (96.9%) were *msp1α*-positive and all were *msp4*-positive. Ten new *msp1α* and five previously reported short-sequence repeats were identified, while *msp4* analysis revealed 18 genotypes, with Somali isolates clustering alongside strains from Africa and Russia.

**Conclusions:**

This study confirms the high prevalence and genetic diversity of *A. marginale* in Somali cattle, highlighting the need for improved surveillance and control measures.

**Graphical Abstract:**

**Figure:**
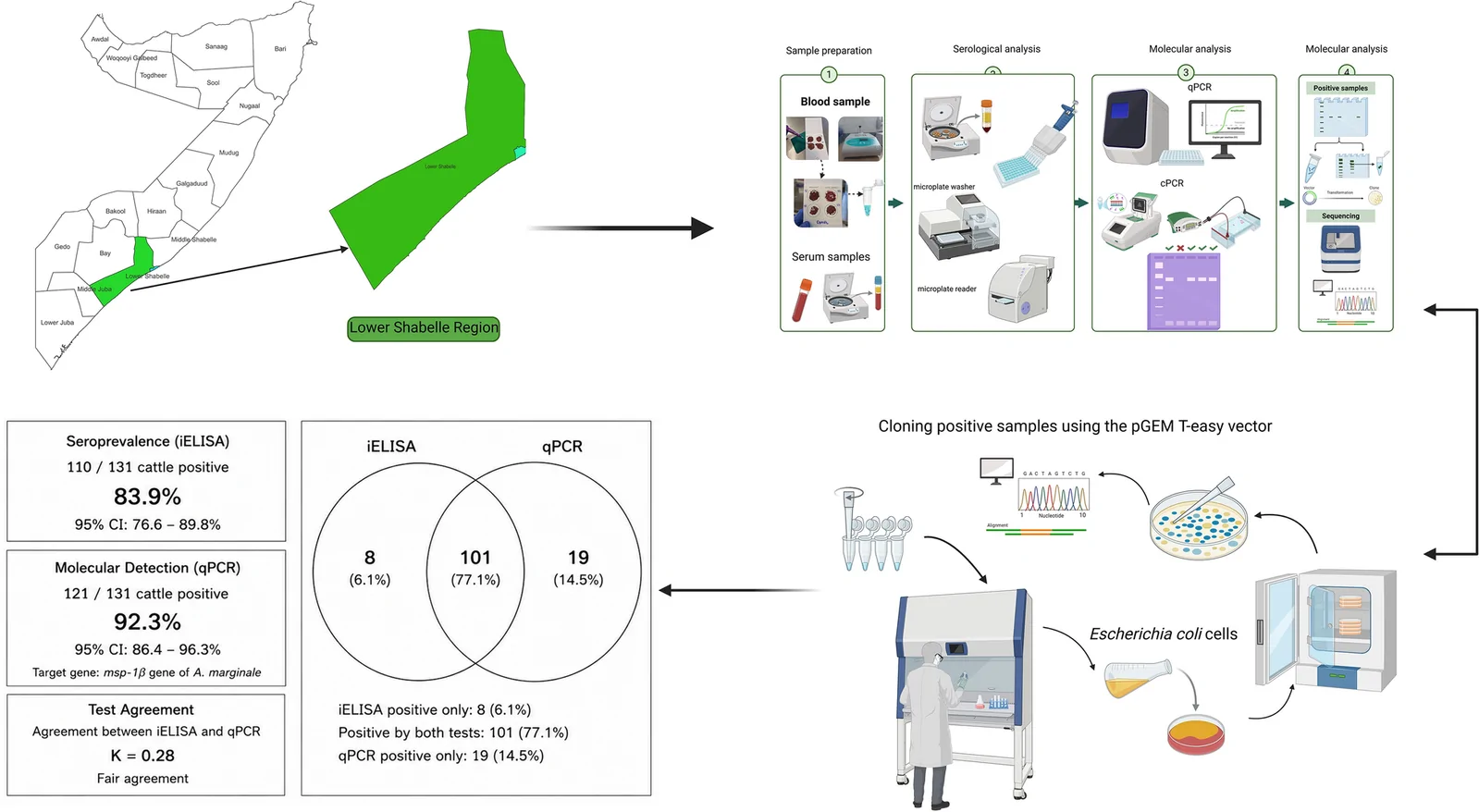

**Supplementary Information:**

The online version contains supplementary material available at https://doi.org/10.1186/s13071-026-07563-2.

## Background

Tick-borne diseases (TBDs) are a major constraint on the growth of the livestock industry around the world [1], posing a significant and increasing economic impact each year [2]. Tick-borne diseases affect 80% of the world’s cattle population, with an estimated cost between USD 13.9 billion and USD 18.7 billion [3]. In Africa, TBDs represent a critical animal health concern, especially for smallholder farmers across the eastern, central, and southern regions [4]. In Somalia, the tropical climate is ideal for animal production, but it also supports large tick populations, which may increase TBD transmission. In a recent study, ticks collected from cattle were identified as *Rhipicephalus pulchellus, Amblyomma gemma, Amblyomma lepidum, Hyalomma marginatum, Hyalomma rufipes*, and *Rhipicephalus pravus* [5].

*Anaplasma marginale* (Rickettsiales: Anaplasmataceae) is an obligate intracellular tick-borne alphaproteobacterium that parasitizes erythrocytes [6]. It is the etiological agent of bovine anaplasmosis worldwide, resulting in significant economic losses for the cattle industry due to reduced meat and milk yields and high mortality rates in various climates [6, 7]. Clinical disease is most commonly observed in cattle older than one year of age. The predominant clinical signs include severe anemia, jaundice (without hemoglobinuria), fever, weight loss, lethargy, depression, and occasional abortion [8]. *Anaplasma marginale* is transmitted biologically by ticks of the genus *Rhipicephalus* spp. in tropical regions and *Dermacentor* spp. in temperate regions [6], mechanically by other hematophagous arthropods, including horseflies (*Tabanus* spp.) and stable flies (*Stomoxys calcitrans*), as well as through blood-contaminated fomites and transplacental transmission [6, 7, 9].

Knowledge of the genetic diversity of *A. marginale* strains is important for epidemiological and virulence studies and control strategies [10]. The genetic diversity of *A. marginale* has been characterized based on sequencing major surface proteins (MSPs), like Msp1a, Msp1β, Msp4 and Msp5 [11]. Major surface protein 4 (MSP4) has been used in phylogeographic studies, while the conserved nature of MSP5 makes it suitable as an antigenic substrate for serological diagnosis. On the other hand, Msp1a is a marker for *A. marginale* genetic diversity and strain distinctions [12].

The analysis of the *msp1α* gene in *A. marginale* offers insights into genetic diversity, host–pathogen evolution, and transmission by ticks [13, 14]. It aids in comparing strains within regions, informs vaccine design, and serves as a stable genetic marker for epidemiological studies, as the gene remains conserved during infections in both cattle and ticks, despite the variability of its tandem repeat sequences [8, 15–17].

In Somalia, conventional techniques for detecting bovine anaplasmosis, notably microscopic assessment of stained blood smears, have traditionally been employed for identifying *Anaplasma* spp. [18, 19]. Unfortunately, this approach exhibits limitations in terms of sensitivity and specificity. Despite the importance of livestock to Somalia’s economy, serological and molecular techniques for diagnosing bovine anaplasmosis remain largely unexplored in the region, highlighting a gap in current knowledge. Therefore, this study aimed to investigate (i) the serological and molecular occurrence of *A. marginale*, and (ii) the genetic diversity of *A. marginale* based on tandem repeats, microsatellites, and haplotype analyses of the *msp1α* and *msp4* genes.

## Methods

### Study area and design

This study was conducted in Lower Shabelle, a region in southern Somalia known for its strong livestock and agricultural sectors. Livestock farming is a key pillar of the regional economy, with cattle representing the predominant livestock species. The region features a semi-arid to arid climate with distinct wet and dry seasons. The wet seasons typically occur from April to June and from October to November, bringing moderate to heavy rainfall. The dry season extends from December to March.

**Fig. 1:**
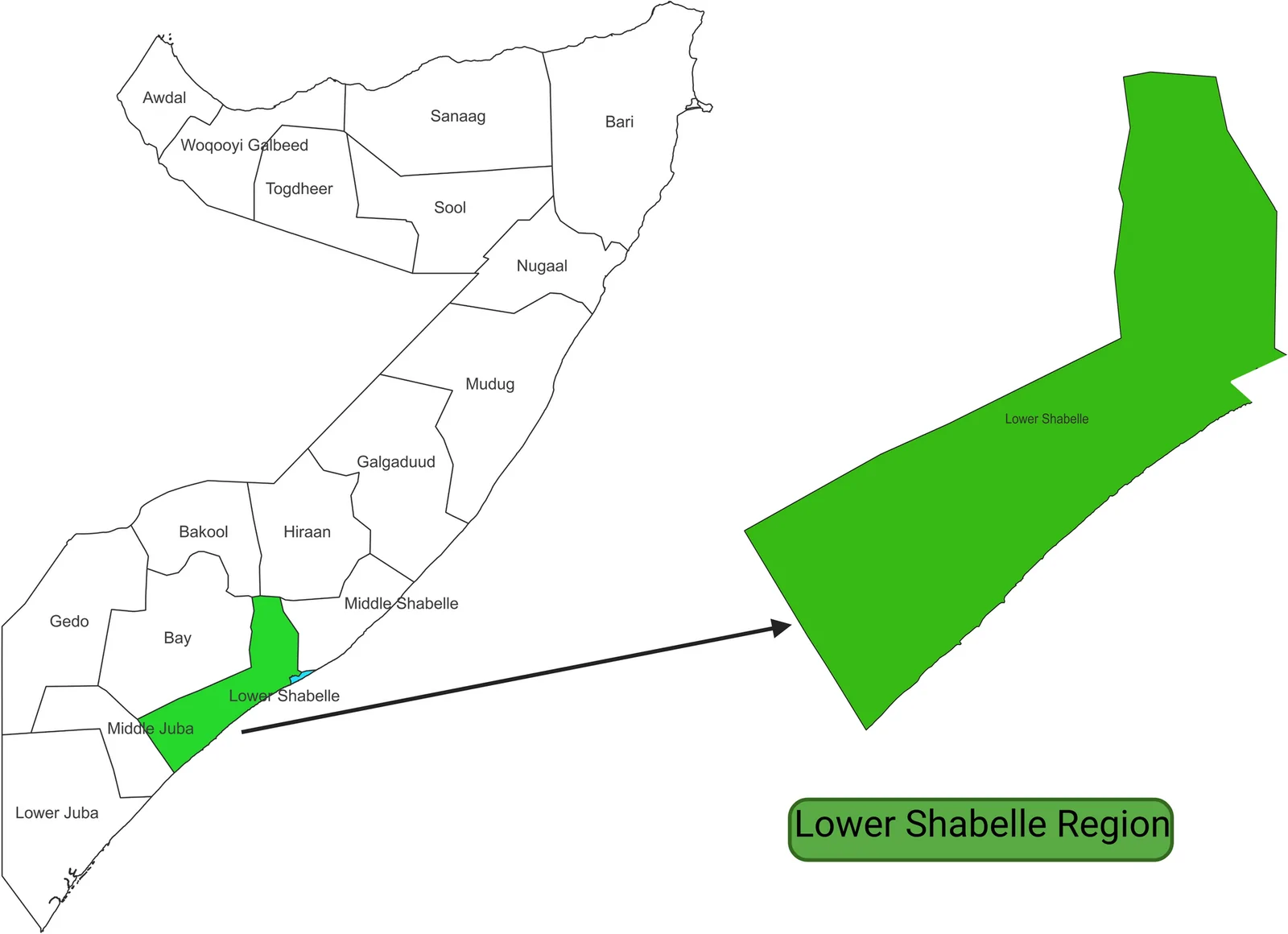
Map of Somalia showing the sampling sites. The highlighted green (Lower Shabelle region) indicate the location of the sampled area. The figure was generated and modified using QGIS software version 3.26.0

A cross-sectional study was carried out during the dry season, from November 2019 to January 2020, in three districts of Lower Shabelle: Wanla Weyn (2°08′17.16″N, 45°07′16.32″E), Awdhegle (1.98050°N, 44.83300°E), and Afgoye (2°08′47.67″N, 45°07′08.11″E) (Fig. 1). A non-probabilistic convenience sampling method was used due to logistical constraints and field accessibility. Given the non-probabilistic nature of sampling, the study should be interpreted as an exploratory field investigation. The results should be interpreted as descriptive of the sampled population. Cattle were recruited from 11 farms (median of 12 animals per farm; SD ± 5.4). Farms were selected based on owners’ willingness to participate and the feasibility of access at the time of fieldwork. Within each farm, animals were sampled opportunistically from available herds without prior stratification. Blood samples were collected from 131 cattle (*Bos taurus indicus*) (122 females and nine males), distributed across Awdhegle (*n* = 64), Wanla Weyn (*n* = 48), and Afgoye (*n* = 19).

Cattle were physically restrained, and approximately five mL of blood were collected from the jugular vein using EDTA-containing vacuum tubes (BD Vacutainer^®^, Franklin Lakes, NJ, USA) for molecular analysis and packed cell volume (PCV) measurement. Additional five mL of blood were collected into tubes containing serum separator gel (BD Vacutainer^®^, Franklin Lakes, NJ, USA) and kept at room temperature (25 °C) until clot formation. The samples were then centrifuged at 1,500 × g for 5 min, and the serum was separated and stored at − 20 °C for serological analysis. These samples had previously been screened for hemoplasmas [4], *Bartonella* spp. [20], *Ehrlichia* spp. [21], *Coxiella burnetii* [22], and *Rickettsia* spp. [23].

An epidemiological questionnaire was administered to each farm owner to gather information on animal characteristics, including sex, body condition, and tick infestation status. Body condition was assessed using the scoring system described by Nicholson and Butterworth (1986) [24] and classified as poor, medium, or good. Tick samples collected from the same animals involved in this study were previously reported by Ferrari et al. (2022) [5].

### Packed cell volume

Of the 131 animals sampled in this study, packed cell volume (PCV) was successfully determined in 125 animals using the microhematocrit centrifugation technique [25]. A PCV of 0.26 L/L or less was used as an indicator of anemia [26].

### Detection of IgG antibodies against *A. marginale* rMSP5

Cattle serum samples were screened for anti-*A. marginale* IgG antibodies using a commercial rMSP5-based indirect enzyme-linked immunosorbent assay (iELISA) (Imunodot Diagnostics^®^, Jaboticabal, São Paulo, Brazil), following a protocol described elsewhere [27]. The plates (Maxisorp^®^; Nunc, Thermo Scientific, Brazil) containing rMSP5 at a concentration of 2.5 μg/mL in 0.05 M sodium carbonate-bicarbonate buffer (pH 9.6) were incubated in a moist chamber at 37 ºC for 90 min. After three washes with PBS-Tween 20 buffer (pH 7.2), the positive and negative reference serum and tested serum samples, previously diluted (1:100) with PBS-Tween 20 solution plus 5% normal rabbit serum, were added to the ELISA plates. The plates were incubated again at 37 ºC for 90 min. After three washes with PBS-Tween 20 buffer, Rabbit anti-bovine IgG (KPL SureBlue^™^, SeraCare Life Sciences, Milford, USA) alkaline phosphatase conjugate was added to the ELISA plates at a 1:50,000 dilution in PBS-Tween 20 plus 5% of normal rabbit serum, followed by incubation and washing. Finally, the substrate of the alkaline phosphatase enzyme, P-nitrophenyl phosphate (Sigma^®^, St. Louis, MO, USA) diluted to 1 mg/mL in diethanolamine buffer pH 9.8 (Sigma^®^, St. Louis, USA) was added. The ELISA plates were sealed with aluminum foil and incubated at room temperature for 30 min. The reading was performed using ELISA reader Dynex MRX TC Microplate Reader (Dynex Technologies, Shirley, New York, USA), at 405 nm filter. Negative control sera were obtained from colostrum-deprived newborn calves confirmed negative for *A. marginale* by both qPCR and serology. Positive control sera were sourced from naturally infected cattle verified by qPCR and serology. The cutoff values were calculated as 2.5 times the mean absorbance of the negative control sera [27].

### DNA extraction

DNA was extracted from EDTA-whole blood cattle samples using a commercial kit (Illustra^®^ Blood Genomic Prep Mini Spin Kit, GE Healthcare, Little Chalfont, UK), according to the manufacturer's instructions. Ultra-pure water was used as a negative control to monitor cross-contamination in each batch of extractions.

### Polymerase chain reaction (PCR) assays for *A. marginale*

DNA samples extracted from cattle blood were subjected to a conventional PCR for the mammalian glyceraldehyde-3-phosphate dehydrogenase (*gapdh*) endogenous gene [28], to ensure successful DNA extraction. Following this, DNA samples were subjected to a quantitative real-time PCR (qPCR) for *A. marginale* based on the *msp1β* gene, as previously described [29]. Each sample was tested in duplicate in a total reaction volume of 10 μL. The reaction mix contained 1 μL of DNA template, 0.2 μM of an oligonucleotide hydrolysis probe, 0.9 μM of each primer (Table 1), and 5 μL of GoTaq^®^ Probe qPCR Master Mix (Promega^®^, Madison, Wisconsin, USA), with the volume completed using nuclease-free ultrapure water (Promega^®^, Madison, Wisconsin, USA). Amplification was carried out in low-profile Multiplate plates using the QuantStudio™ 5 real-time PCR system (Thermo Fisher Scientific^®^, Waltham, Massachusetts, USA). A standard curve was generated using serial dilutions of a synthetic *msp1β* gene fragment (Integrated DNA Technologies, Coralville, IA, USA). All experimental procedures and analytical parameters adhered to the Minimum Information for Publication of Quantitative Real-Time PCR Experiments (MIQE) guidelines [32]. Amplification efficiency (E) was calculated from the slope of the standard curve in each run using the following formula (*E* = 10 ^−1/slope^) (Bustin et al. 2009). The average efficiency, slope, *R*^2^, and y-intercept are reported. All DNA samples were initially tested in duplicates. Samples showing a Cq difference > 0.5 between duplicates were retested in triplicate. Samples with a median Cq value < 38 were considered positive based on previous validation [29].

**Table 1:** Description of primers, amplicon sizes and thermal conditions used in PCR assays for *A. marginale*

Gene	Primer	Sequence (5′-3′)	Thermal conditions (°C)	Base pair (pb)	References
*msp*1β	AM-F:	TTGGCAAGGCAGCAGCTT	95 °C for 10 min and 40 cycles of 15 s at 95 °C and 1 min at 60 °C	~ 95	[29]
	AM-R:	TTCCGCGAGCATGTTGCAT			
	AM-probe:	6FAM-5′-TCGGTCTAACATCTCCAGGCTTTCAT-3′-BHQ1			
*msp*1α	*msp1α*F1	GTG CTT ATG GCA GAC ATT TCC	94 °C for 5 min; 30 cycles of 94 °C for 30 s, 57 °C for 30 s and 72 °C for 35 s; 72 °C for 7 min	~ 643	[30]
	*msp1α*R^1,2^	CTC AAC ACT CGC AAC CTT GG			
	*msp1α*NF^2^	CGC ATT ACA CGT TCC GTA TG	94 °C for 5 min; 30 cycles of 94 °C for 35 s, 65 °C for 58 s and 72 °C for 30 s; 72 °C for 10 min		
*msp* 4	MSP45	GGGAGCTCCTATGAATTACAGAGAATTGTTTAC	94 °C for 30 s, 35n cycles 94 °C for 30 s, 60 °C for 30 s and 68 °C for 1 min	~ 870	[31]
	MSP43	CCGGATCCTTAGCTGAACAGGAATCTTGC			

^1^First reaction

^2^Second reaction

A total of 32 positive samples for *A. marginale* by the qPCR assay with low Cq values (17.3–25) were prioritized to maximize sequencing success, indicative of higher bacterial loads, were subjected to a PCR assay for *msp4* as previously described [31], and a semi-nested (sn)PCR assay based on the *msp1α* gene [30], to maximize the likelihood of successful amplification and high-quality sequencing. The cycling conditions and primers used in the abovementioned PCR assays are described in Table 1. DNA from *A. marginale* (Jaboticabal strain) was used as a positive control, and sterile ultrapure water (Nuclease-Free Water, Promega Corporation, Madison, WI, USA) was used as a negative control for conventional PCR assays targeting the *msp4* and *msp1α* genes.

### Cloning

Five selected *msp1α* amplicons (cattle ID 91, 112, 114, 123, and 132) that produced strong PCR bands were cloned into the pGEM^®^-T Easy Vector (Promega^®^; Madison, Wisconsin, USA), following the manufacturer’s recommendations. The ligation reaction products were used to transform One Shot™ Mach1™ T1R chemically competent *Escherichia coli* cells (Invitrogen Cat # C8620–03) (10^9^–10^10^ CFU/ng DNA), as previously described [33]. Plates were prepared using LB (Luria Bertani medium—Tryptone; Yeast Extract; NaCl; distilled water q.s.p. (ThermoFisher Scientific, Waltham, MA, USA) agar medium, supplemented with 100 μg/mL ampicillin, 40 μL X-gal (0.026%; 5-bromo-4-chloro-3-indolyl-β-D-galactoside), and 20 μL IPTG (0.82 mM; isopropylthio-β-D-galactoside) to enable blue/white colony screening for successful recombinants. Plasmid DNA was extracted from positive clones using the Wizard^®^ Plus SV Minipreps DNA Purification System (Promega^®^, Madison, Wisconsin, USA), following the manufacturer’s protocol. Purified plasmids were stored at − 20 °C for downstream applications. To confirm successful cloning, plasmid DNA was amplified by PCR using M13 universal primers: M13-F (5′-CGCCAGGGTTTTCCCAGTCACGAC-3′) and M13-R (5′-GTCATAGCTGTTTCCTGTGTGA-3′), as described elsewhere [34].

### Electrophoresis, amplicon purification and Sanger sequencing

The amplified products were separated by electrophoresis in 1% agarose gels. These gels were subsequently stained using ethidium bromide (Life Technologies^™^, Carlsbad, CA, USA), followed by electrophoresis at 90 V for 50 min. The resulting agarose gels were then exposed to ultraviolet light using the ChemiDoc^™^ MP Imaging System (BioRad^®^) and were captured using Image Lab Software version 4.1.

The PCR products generated in the amplification of fragments of the *msp1α* and *msp4* genes were purified using a commercial kit (Promega Wizard^®^ PCR and Gel Clean-Up, Promega^®^; Madison, Wisconsin, USA). Amplicons of *msp4* were sequenced in both directions by Sanger sequencing [35] using the same PCR primers (forward and reverse). Sequencing of cloned *msp1α* fragments was performed using universal primers M13-F (5′-CGCCAGGGTTTTCCCAGTCACGAC-3′) and M13-R (5′-GTCATAGCTGTTTCCTGTGTGA-3′). Quantification of purified DNA was performed using a NanoDrop™ 2000c spectrophotometer. The amplicons were sequenced at the Center for Human Genome and Stem Cell Studies Institute of Biosciences – University of São Paulo, SP, Brazil. The obtained sequences were compared with those available in the GenBank^®^ database using the BLASTn tool (National Center for Biotechnology Information, Bethesda, MD, USA) [36].

### Bioinformatic analyses

The *msp1α* nucleotide sequences of of *A. marginale* identified in this study were compared with those already available in the GenBank^®^ database to analyze the *msp1α* genetic diversity. A microsatellite was observed at the 5′ UTR of the *msp1*α gene between the Shine-Dalgarno sequence (GTAGG) and the translation initiation codon (ATG) [15]. Microsatellite structures were characterized as GTAGG (G/A TTT) m (GT) n ATG [37]. The SD-ATG distance was calculated using the formula (4 × m) + (2 × n) + 1, as described elsewhere [37].

The analysis of *msp1α* genotypes was performed according to the nomenclature proposed by Allred et al. (1990). The RepeatAnalyzer software was used to identify, manage, and analyze tandem repeats of *A. marginale msp1α* [38]. Genetic diversity was assessed using metric indices that quantify the proportion and distribution consistency of single repeat sequences within the study area. The software determined the frequency of each short-sequence repeats (SSRs) in the study area and highlighted distinctive strains unique to this study, distinguishing them from those in the database. Novel SSRs were defined as those not present in the RepeatAnalyzer database or in any previously published *msp1α* gene sequences. New SSRs were assigned identifiers SO1–SO10, where 'SO' denotes Somalia.

An alignment with total size of 596 bp was constructed for the *msp4* gene using MAFFT [39] including the six sequences obtained in the present study alongside 64 homologous sequences from *A. marginale msp4* gene deposited in GenBank^®^ database. Values of nucleotide diversity (π), polymorphism level (haplotype diversity—[dh]), number of genotypes (h) and the average number of nucleotide differences (K) between the sequences were obtained by DnaSP program v5 [40]. The haplotype network was constructed using PopART (http://popart.otago.ac.nz), and the TCS inference method [41]. Additionally, a distance-based analysis was performed using SplitsTree v4.14.6 [42] to further investigate the genetic relationship among *msp4* sequences from the present study and 37 homologous sequences previously deposited in GenBank^®^ database, based on a Neighbor-Joining network.

The gene sequences obtained herein were submitted to the GenBank^®^ database under the following accession numbers: for *msp1α* gene (PX055668–PX055678), and for *msp4* gene extended fragment (PV012670–PV012675).

### Statistical analysis

The Shapiro–Wilk normality test was conducted to assess the distribution of the PCV data. Therefore, a non-parametric Mann–Whitney test was used to compare the PCV concentration between infected and non-infected cattle. Univariable Chi-square test were used to determine individual variables like tick presence, body condition, anemia, and location with *A. marginale* infection. Odds ratio (OR), 95% confidence intervals (95% CI), and *P*-values were calculated for each variable. These results were interpreted cautiously due to potential confounding and intra-farm clustering. Statistical significant was set at *P* ≤ 0.05. Due to the limited number of farms (*n* = 11), multivariable analyses accounting for clustered sampling were not feasible. Univariable analyses were presented as descriptive associations, and intra-farm correlation was not adjusted for. Data were compiled and analyzed in Epi Info™ software, version 7.2.3.1 (Centers for Disease Control and Prevention, CDC, USA). The kappa coefficient (*κ*) of agreement between the serological test and qPCR assay was calculated using freely available software (http://www.openepi.com). The magnitude of κ coefficients was interpreted as follows: ≤ 0 poor, 0.01–0.2 slight, 0.21–0.4 fair, 0.41–0.6 moderate, 0.61–0.8 substantial, and 0.81–1 almost perfect agreement [43].

## Results

The overall individual animal-level seroprevalence of anti-*A. marginale* IgG antibodies was 110/131 (83.9%; 95% CI 76.6–89.8%). The *gapdh* gene was consistently amplified from all 131 cattle DNA samples and 121/131 (92.3%; 95% CI 86.4 – 96.3%) animals tested positive by qPCR for *A. marginale*. The qPCR performance showed an average efficiency of 96.1% (SD ± 6.5), with an *R*^2^ of 0.85 (SD ± 0.12), a slope of − 3.378 (SD ± 0.162), and a y-intercept of 36.2 (SD ± 3).

The PCV data were not normally distributed (Shapiro–Wilk normality test, *W* = 0.98, *P* = 0.025). The median PCV concentration for cattle was 0.28 L/L. A total of 60/125 (48%, 95% CI38.9–57.1%) cattle were anemic. A statistically significant difference (U = 720.5, Z =  − 2.21, *P* = 0.026) was found in median PCV between *Anaplasma*-seropositive (0.26 L/L) and *Anaplasma*-seronegative cattle (0.30 L/L). However, no significant difference was detected in PCV levels based on the presence/absence of *A. marginale* DNA assessed by qPCR targeting the *msp1β* gene.

Sampling location was associated with *A. marginale* seroprevalence. iELISA results showed the highest seroprevalence in Awdhegle (95.3%), followed by Wanla Weyn (77.1%) and Afgoye (63.2%), with statistically significant differences (*P* = 0.005). Cattle from Awdhegle were six times more likely to be seropositive compared to those from Wanla Weyn (OR = 6.0). qPCR results showed a similar trend: Awdhegle (96.9%), Wanla Weyn (87.5%), and Afgoye (89.5%) though these differences were not statistically significant (*P* = 0.07). Regarding body condition, animals in poor to moderate body condition had a slightly higher seroprevalence (86.7%) and qPCR positivity (95%) compared to those in good condition (81.7% and 90.1%, respectively), though these differences were not statistically significant. Likewise, both iELISA and qPCR results showed similar infection rates in cattle with and without ticks, with no statistically significant differences observed (*P* = 0.69 and *P* = 0.29, respectively) (Table 2).

**Table 2:** Prevalence of cattle *Anaplasma marginale* within each variable studied

Variable		iELISA *A. marginale rMSP5*				qPCR *msp1β* gene			
		+ /n	Prevalence (%) (95% CI)	*P*-value	OR (95% CI)	+ /n	Prevalence (%) (95% CI)	*P*-value	OR (95% CI)
Location	Awdhegle	61/64	95.3 (86.9–99.0)	0.005 (*χ*^2^ = 8.3)	6.0 (1.9–23.1)	62/64	96.9 (89.2–99.6)	0.07 (*χ*^2^ = 3.6)	4.4 (0.9–23)
	Afgoye	12/19	63.2 (38.4–83.7)	0.27 (*χ*^2^ = 1.3)	0.5 (0.2–1.9)	17/19	89.5 (66.9–98.7)	0.86 (*χ*^2^ = 0.05)	1.2 (0.2–6.6)
	Wanla Weyn	37/48	77.1 (62.7–87.9)	ref		42/48	87.5 (74.8–95.3)	ref	
Body condition	Poor/moderate	52/60	86.7 (75.4–94.1)	0.45 (*χ*^2^ = 0.6)	1.5 (0.6–3.8)	57/60	95 (86.1–98.9)	0.32 (*χ*^2^ = 1.1)	2.1 (0.5–8.4)
	Good	58/71	81.7 (70.7–89.9)	ref		64/71	90.1 (80.7–95.9)	ref	
Tick presence	Yes	27/33	81.8 (64.5–93.0)	0.69 (*χ*^2^ = 0.15)	0.8 (0.3–2.3)	29/33	87.8 (71.8–96.6))	0.29 (*χ*^2^ = 1.26)	0.5 (0.1–1.8)
	No	83/98	84.7 (76.0–91.2)	ref		92/98	93.9 (87.2–97.7))	ref	

+ , number of positive animals; *n*, number of samples; 95% CI, 95% confidence interval; OR, odds ratio; ref, Reference group

**Fig. 2:**
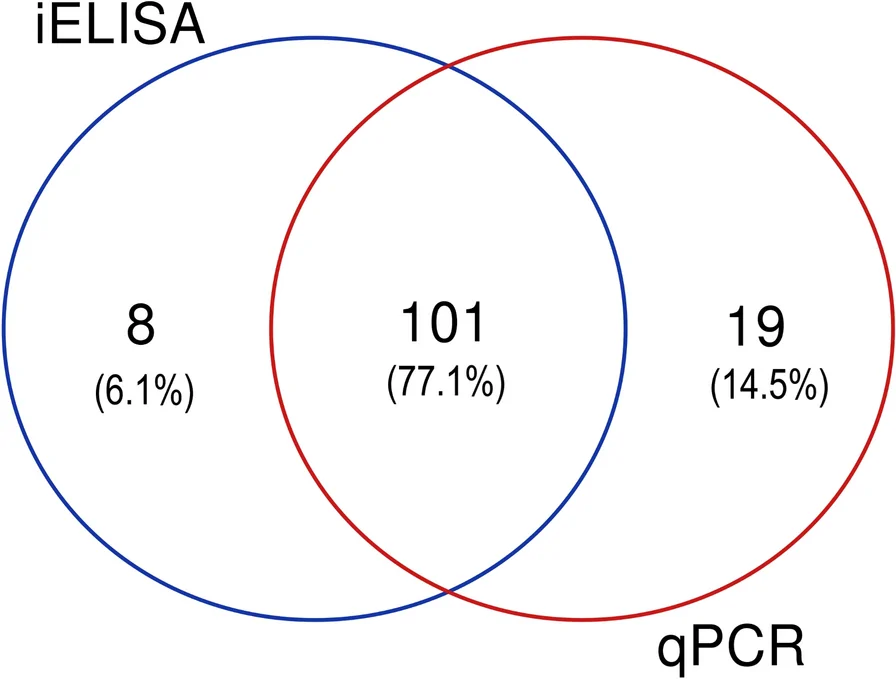
Venn diagram illustrating the overlap and unique detection of *Anaplasma marginale* by qPCR and iELISA. Values represent the number of animal samples testing positive by each method or their combination, while percentages are calculated based on the total number of animals sampled (*n* = 131)

The agreement between the commercial iELISA test and qPCR based on the *msp1β* gene of *A. marginale* was fair (*k* = 0.28). Figure 2 presents a Venn diagram comparing the detection of *A. marginale* using iELISA and qPCR.

A total of 32 *A. marginale* qPCR-positive samples with Cq values ranging from 17.3 to 25 were selected for genetic diversity analysis. Out of these, all tested positive for the *msp4* gene, while 31/32 (96.8%; 95% CI 83.3–99.9%) tested positive for the *msp1α* gene.

The analysis of the *msp1α* gene repeat region structure revealed ten new and five previously reported SSRs in cattle from Somalia. Of these SSRs, eight (53.3%) consisted of 29 amino acids, while seven (46.7%) comprised 28 amino acids (Table 3). Notably, the most prevalent SSR observed was "81", found in eight samples. Nine distinct strains were detected from five cattle samples, with all of the strains contained novel genotypes (Table 4). Animals 112 and 123 were each infected with two strains, while animal 114 was infected with three strains. Interestingly, the number of Msp1a SSR displayed variation across distinct *A. marginale* strains, ranging from three to five repeats. The most commonly observed strains were those with four (36.4%), followed by three (27.3%), and five (27.3%). The microsatellite in the 5′ untranslated region (UTR) of the *msp1α* gene revealed infection with two genotypes (E and G) (Table 4).

**Table 3:** Sequences of *msp1α* tandem repeats found in *A. marginale* isolates from Somalia

Repeat form	Encoded sequence	Number of Amino acids
	*	
A	DDSSSASGQQQESSVSSQS-EASTSSQLG	28
E	ADSSSASGQQQESSVSSQS-EASTSSQLG	28
72;80	ADSSSAGDQQQESSVSSQSGQASTSSQLG	29
81	TDSSSAGDQQQESSVSSQSGQASTSSQLG	29
98	ADSSSASGQQQESSVLSQS-EASTSSQLG	28
106	ADGSSAGDQQQESSVSSQSGQASTSSQLG	29
SO1	ADSSSAGDQQQESSVSSQGGQASTSSQLG	29
SO2	TDSSSAGDQQQEGSVSSQSGQACTSSQLG	29
SO3	TDSSSAGDQQQESSVSTQSGQASTSSQLG	29
SO4	ADSSSVGDQQQESSVSSQSGQASTSSQLG	29
SO5	TDSSSAGDQQQESSVSSQGGQASTSSQLG	29
SO6	ADSSSASGLQQESSVLSQS-EASTSSQLG	28
SO7	ADSPSASGQQQEGSVLSQS-EASTSSQLG	28
SO8	ADSSSASGQQQEGSVLSQS-EASTSSQLG	28
SO9	ADSSSAGGQQQEGSVLSQS-EASASSQLG	28
SO10	ADSTSASGQQQEGSVLSQS-EASTSSQLG	28

The one-letter amino acid code depicts the *msp1α* repeats. * Indicates position 20. Repeat A is used as a reference, with amino acids identical to repeat A in black, those that differ are red and dashes indicate deletions/insertions with respect to the reference repeat [44]

**Table 4:** Structure of the *msp1α* microsatellite sequences of 11 *A. marginale* isolates from cattle from Somalia

Animal ID	Clone ID	GenBank accession number	Microsatellite genotype	m^a^	n^b^	SD-ATG distance	Number of Repeats	*msp1α* Genotype				
91	2	PX055668	G	3	5	23	3	SO1	81	81		
91	3	PX055669	E	2	7	23	3	SO1	81	81		
112	1	PX055670	G	3	5	23	4	106	81	81	SO2	
112	2	PX055671	G	3	5	23	4	106	81	81	SO2	
112	6	PX055672	G	3	5	23	4	106	SO3	81	81	
114	2	PX055673	G	3	5	23	4	72;80	81	81	81	81
114	5	PX055674	G	3	5	23	4	72;80	81	81	81	
114	6	PX055675	G	3	5	23	4	SO4	SO5	81	81	
123	2	PX055676	G	3	5	23	5	SO6	SO7	98	SO10	98
123	5	PX055677	G	3	5	23	5	98	SO8	98	SO9	98
132	1	PX055678	E	2	7	23	5	98	98	98	98	E

The microsatellite was located between the Shine-Dalgarno and the translation initiation codon (ATG) with the structure: GTAG/AG (G/ATTT) m (GT)n T ATG. The SD-ATG distance was calculated in nucleotides as (4 × m) + (2 × n) + 1

m^a^ is the number of repetitions of the nucleotide sequence G/ATTT

n^b^ is the number of repetitions of the nucleotide sequence GT

The *msp4* (~ 596 bp) *Anaplasma* spp. gene sequences from six samples showed 99.73–100% identity (E-value = 0.0; query cover = 100%) to multiple *A. marginale msp4* gene sequences deposited in the GenBank^®^ database (OP649851, MH191396, MW273291, AY666010, OK663651, KY362505, KX989521, KU497715, KF739431, and AY666005), described in cattle from Algeria, Russia, Kenya, Zimbabwe, Nigeria, Tunisia, India, Sudan, Italy, and South Africa, respectively.

**Fig. 3:**
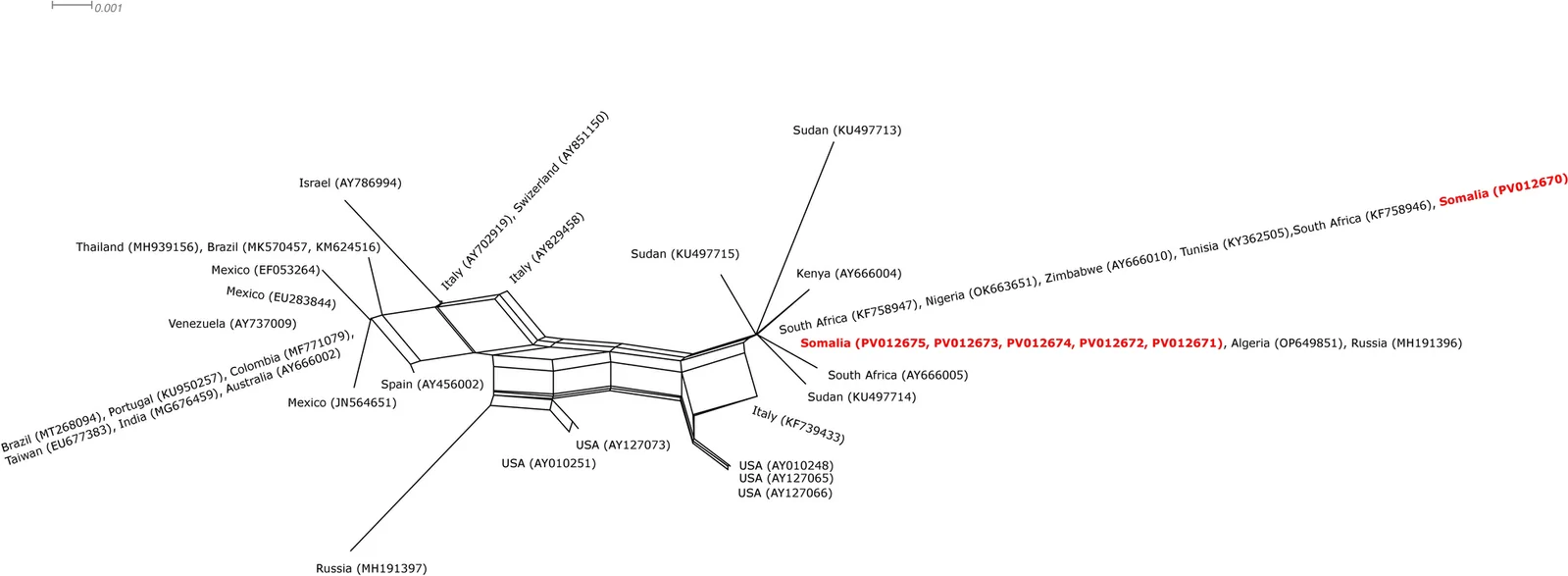
Distance analysis using the Splitstree software with the “Neighbor-Net” and “Uncorrected p-distance” parameters. Sequences of the *Anaplasma marginale msp4* gene from Somalia, grouped with sequences from Algeria, South Africa, Nigeria, Zimbabwe, Tunisia, Russia. Sequences obtained in the present study are highlighted in red. All other sequences were retrieved from GenBank^®^ database

**Fig. 4:**
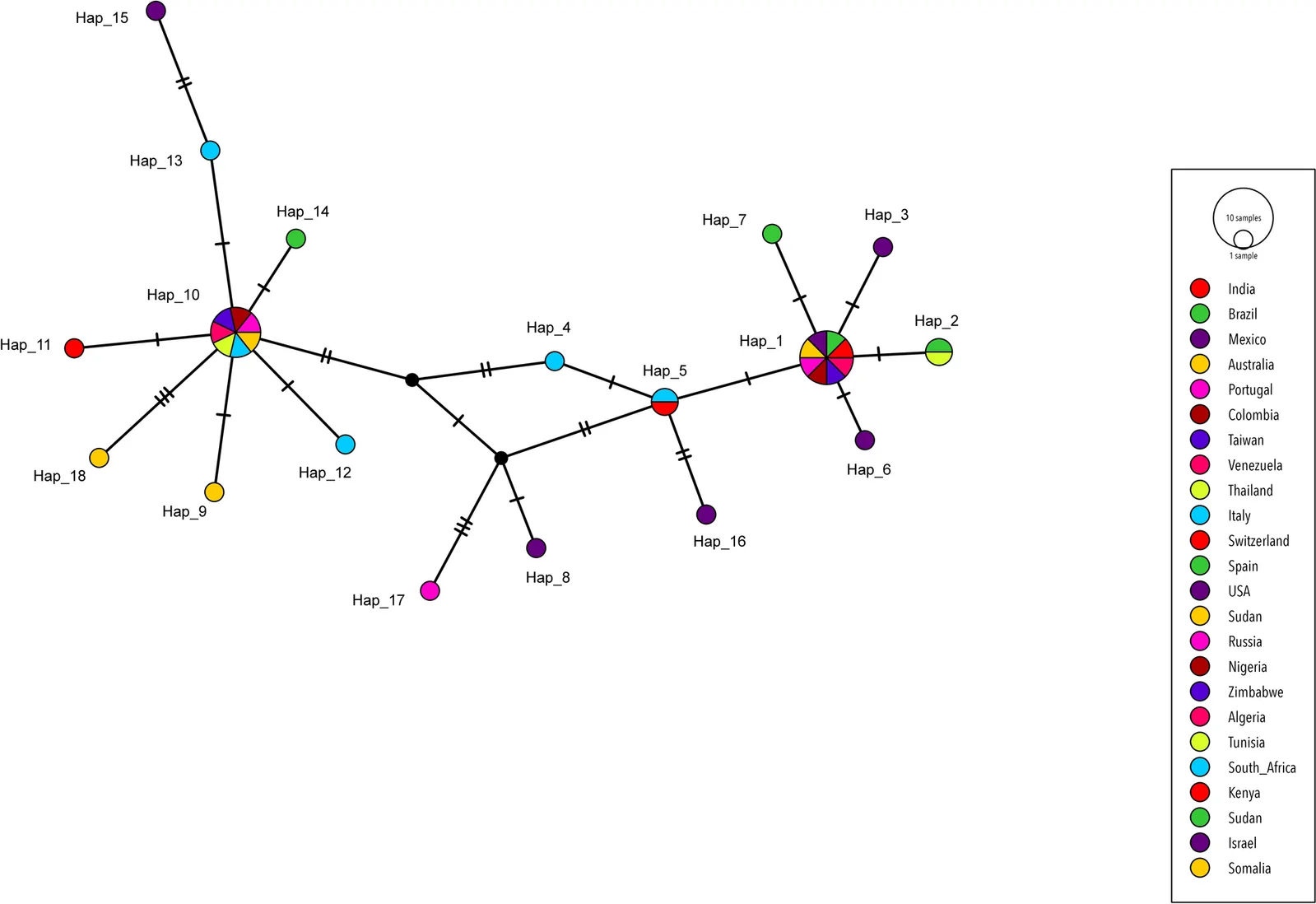
A network of *Anaplasma marginale* msp4 genotypes (Hap) detected in cattle sampled in Somalia, together with those obtained from the GenBank^®^ database. Black spots represent median-vectors. Small traits between one identified haplotype and another represent mutational events. The *A. marginale msp4* sequences grouped within genotype #10

The diversity analysis for the *msp4* gene dataset included sequences generated in this study and reference sequences retrieved from GenBank^®^ database. The analysis identified a total of 18 haplotypes (h) with a haplotype diversity (hd) of 0.731 ± 0.050 and a nucleotide diversity (π) of 0.00583 ± 0.00049. The average number of nucleotide differences (k) between sequences was 3.47453. Sequences from the present study clustered within haplotype number 10 (Hap_10), along with sequences from Russia (MH191396), Nigeria (OK663651), Zimbabwe (AY666010), Algeria (OP649851), Tunisia (KY362505), and South Africa (KF758946) (Fig. 3). Table 5 provides the countries of origin and GenBank accession numbers of the sequences representing each haplotype identified in this study. SplitsTree analysis showed a similar pattern, with sequences from Somalia clustering closely with those in Hap_10. Furthermore, sequences obtained from various African localities were positioned closely in this analysis, along with the Russian sequence (MH191396) (Fig. 4).

**Table 5:** Description of countries of origin and GenBank® database access number of *Anaplasma marginale msp4* sequences representing each hapotyple identified in the present analysis

Variant	Country	GenBank® access number
Hap_1	India, Brazil, Mexico, Australia, Portugal, Colombia, Taiwan, Venezuela	KX989520, MK570467, MK570465, MT268094, MK570458, MK570462, MK570463, MK570461, MK570456, JN564647, KX989516, MK570468, MT683170, AY714546, AY666002, KU950257, MK570464, MK570459, MK570460, KX989519, KX989518, MK570469, MT683169, EU283845, MG676453, KM624517, MF771078, MF771079, MF771080, EU677383, AF428083, MG676459, EU283844, AY737009
Hap_2	Brazil, Thailand	MK570457, KM624516, MH939156, MH939155
Hap_3	Mexico	JN564651
Hap_4	Italy	AY829458
Hap_5	Italy, Switzerland	AY702919, AY851150
Hap_6	Mexico	EF053264
Hap_7	Spain	AY456002
Hap_8	USA	AY010251, AY127073
Hap_9	Sudan	KU497715
Hap_10	Russia, Nigeria, Zimbabwe, Algeria, Tunisia, South Africa, Somalia	MH191396, OK663651, AY666010, OP649851, KY362505, KF758946, KF758947, **PV012670, PV012671, PV012672, PV012673, PV012674, PV012675**
Hap_11	Kenya	AY666004
Hap_12	South Africa	AY666005
Hap_13	Italy	KF739433
Hap_14	Sudan	KU497714
Hap_15	USA	AY010248, AY127065, AY127066
Hap_16	Israel	AY786994
Hap_17	Russia	MH191397
Hap_18	Sudan	KU497713

Samples from the present study are highlighted in bold

## Discussion

Bovine anaplasmosis caused by *A. marginale* is one of the most important cattle diseases, due to its negative impact on the cattle industry worldwide, resulting in substantial economic losses for livestock producers [6]. Despite its importance, the disease remains underdiagnosed and poorly characterized in several regions, including Somalia.

In this study, iELISA using the recombinant protein MSP5 revealed a high seropositivity rate (83.9%) for *A. marginale* among the animals sampled. This substantial seropositivity for *A. marginale* aligns with previous research conducted in Brazil, where seropositivity rates were reported at 81% [43], and 91.25% [45]. In contrast, a study conducted in the USA reported seropositivity ranging from 19.1% of herds in Western Kansas to 87.3% of herds in Eastern Kansas [46]. However, serological positivity should be interpreted as evidence of exposure rather than active infection, as IgG antibodies against MSP5 can persist for extended periods following infection. It is important to note that MSP5 is a highly conserved protein among *Anaplasma* species, and thus, the potential for serological cross-reactivity cannot be ruled out [47]. Despite this, the high seropositivity observed in the iELISA is corroborated by the qPCR results, which showed a 92.3% molecular detection rate of *A. marginale* among the same samples, further supporting widespread exposure in the studied population. Additionally, the fair agreement observed between iELISA and qPCR (*κ* = 0.28) is biologically and diagnostically expected. These two tests measure different aspects of infection: iELISA reflects historical or cumulative exposure, whereas qPCR detects current infection. Discordant results can therefore be explained by the infection timeline. Animals that were qPCR-positive but seronegative likely represent early-stage infections prior to seroconversion. Conversely, animals that were seropositive but qPCR-negative might have experienced prior exposure with declining or intermittent bacteremia below the detection limit of qPCR. Additionally, in endemic settings, such as those suggested by the high exposure observed in this study, cattle populations may develop endemic stability, characterized by widespread exposure at an early age with limited clinical disease. Under such conditions, high seroprevalence alongside variable qPCR detection is expected, reflecting persistent infection with fluctuating bacteremia levels.

To the best of our knowledge, this study provides the first molecular evidence of *A. marginale* infection in cattle from Somalia. The qPCR technique used herein is highly specific for the *msp1β* gene of *A. marginale*, with no cross-reaction with other known pathogens [29]. A high positivity rate (92.3%) for *A. marginale* was found among the studied animals. This finding is consistent with previous research conducted in Brazil, which reported positivity rates of 95.3% and 100% [45]. However, our results indicate a higher positivity rate than a study conducted in Tunisia, where a rate of 38.2% was reported [48].

The microsatellite analysis of the *msp1α* gene following Estrada-Peña et al. (2009) [37] showed the occurrence of two *A. marginale* genotypes, namely G and E, among Somali cattle. The G genotype was the predominant one, representing nine/11 (81.8%) of the cases, while the E genotype was the least prevalent at two/11 (18.2%). Previous studies carried out in Turkey identified genotypes G, B, and C [49]. In Brazil, genotypes E, C, and B were found among Nelore beef cattle in the Midwestern [50], H, F, E, G in Angus beef cattle in southeastern [45], and genotypes H, E, F, C, B, and G in dairy cattle in the northeastern region of the country [43]. The findings described herein were consistent with previous reports from Brazil [45], Turkey [50], Russia [51], and Uruguay [52], where the G and E genotypes were commonly observed. Genotype G, in particular, stands as the most prevalent genotype worldwide, showing significant prevalence in South Africa and certain regions of the USA and Mexico [37].

Biological transmission of *A. marginale* in tropical and subtropical regions is primarily associated with ticks, namely *Rhipicephalus* spp. (e.g., *R. microplus* and *R. annulatus*) and *Dermacentor* spp. (e.g., *D. andersoni, D. variabilis,* and *D. albipictus*) [53]. However, these tick species have not been reported in Somalia. Tick specimens collected from animals in our study were identified as *R. pulchellus, A. gemma, A. lepidum, H. marginatum, H. rufipes,* and *R. pravus* [5]. Although *Hyalomma* species have been implicated as competent vectors of *A. marginale* in South Africa, their role in transmission within Somalia remains unconfirmed. Therefore, alternative transmission pathways including mechanical transmission via biting dipterans, veterinary instruments, or animal husbandry practices may also contribute to pathogen spread in this setting.

According to de la Fuente et al. (2003) [54], Msp1a repeats with negatively charged amino acids, such as aspartic acid (D) and glutamic acid (E), at position 20 were shown to be essential for binding of Msp1a to the tick cell extract (TCEs), which is a prerequisite for pathogen infection and biological transmission by ticks [54, 55]. Conversely, strains with a G as the 20th amino acid do not exhibit this binding capability [54]. In our study, we found that 50% of the repeats contained a G at the 20th amino acid in an SSR position. While this molecular pattern suggests that a substantial proportion of strains may have reduced potential for biological tick transmission, this inference is based solely on sequence characteristics and was not directly tested in the present study. Functional assays and vector competence studies would be required to confirm whether these strains are indeed less adapted to tick-mediated transmission in Somalia. The predominance of strains carrying a G as the 20th amino acid may be associated with the low occurrence of competent tick vectors in this study area. This molecular profile aligns with our field data, which showed a relatively low prevalence of tick infestation, i.e., in only 30/131 animals (22.90%; 95% CI: 16.54–30.81%) [5]. This figure is notably lower than the overall prevalence of *A. marginale* detected in the same population. Together, these findings suggest that mechanical transmission play an important role in the epidemiology of bovine anaplasmosis in the study region. However, to validate this hypothesis, future research focusing on the distribution of hematophagous dipterans in the studied farms as well as functional characterization of these *A. marginale* strains is warranted. In addition, studies investigating the potential tick vectors of *A. marginale* in Somalia are much needed.

The length of the Msp1a microsatellite is important for the expression of *msp1α* gene, affecting the infection and transmission of *A. marginale*. SD-ATG distances of 23 and 29 nucleotides are associated with higher expression levels compared to a length of 19 nucleotides [37]. In our study, it is noteworthy that 11/11 (100%) of the genotypes showed SD-ATG distances spanning 23 nucleotides, suggesting a potentially high capacity for infection and mechanical transmission of the *A. marginale* strains detected herein.

The genetic variability of *A. marginale* is commonly assessed using the *msp4* and *msp1α* genes [48, 56]. Among these, the *msp4* gene has been proposed as a molecular marker for investigating the phylogeographic patterns of this pathogen [15, 57–59]. In the present study, the *msp4* genotypes identified were found to be clustered to sequences previously reported from various African regions, as well as one sequence from Russia.

The cloning technique employed in this study enabled the identification of multiple *A. marginale* strains within a single animal during a single sampling event. This can arise from co-infection, when two strains are transmitted at the same time or through sequential infection, referred to as superinfection, which explicitly indicates that infection with a second genetically distinct strain occurs after an animal has already developed an immune response to a primary strain, as described by Ueti et al. (2012) [59]. Analysis of the tandem repeat regions, following the classification by Catanese et al. (2016) [38], revealed the presence of up to 15 distinct SSRs (81, 98, 106, 72;80, E and SO1-10) assembled into ten novel genotypes (strains). Three animals appeared to be infected with a single strain, while the remaining two animals were co-infected with two or three strains. These results demonstrate that different strains can coexist within the same ecosystem, indicating the natural occurrence of co-infection or superinfection, which is common in natural infections, as previously reported by Ueti et al. (2012) [60] and Palmer & Brayton (2013) [60]. Furthermore, our findings support the observations of Palmer & Brayton (2013) [60], who noted that several Msp1a SSRs can circulate within a single host, with most animals harboring a dominant SSR while some carry multiple SSRs concurrently. The newly identified SSRs were assigned the identifiers SO1–SO10, where “SO” denotes Somalia, following a location-based nomenclature approach adopted in recent studies to avoid duplication and ensure global traceability of novel repeats. This systematic naming reflects their first detection in Somalia and confirms these SSRs as novel additions to the existing Msp1a repeat database.

We acknowledge certain limitations in our study. The use of convenience sampling from only 11 volunteered farms might have introduced selection bias and limited generalizability, while sampling multiple animals per farm could have created unadjusted clustering. In addition, the lack of detailed animal-level data restricted risk factor analysis. Furthermore, a multivariable analysis was not performed because of the limited number of farms and the clustered sampling design, which constrained our capacity to adequately control confounding and farm-level random effects. Failure to adjust for farm-level clustering may have led to overestimation of precision in association estimates. Additionally, only a limited number of qPCR-positive samples were subjected to genetic characterizing *A. marginale* Msp1a SSRs (*n* = 15) and strains (*n* = 10) and Msp1a microsatellite sequences (*n* = 2) and Msp4 (*n* = 1) genotypes in the Lower Shabelle region of Somalia. Notably, the sampling strategy was not probability-based, which limits the generalizability of our findings to other cattle populations across the country. Additionally, only a limited number of positive samples were subjected to sequencing, which may not fully capture the genetic diversity of *A. marginale* circulating in the region. Although the rMSP5-based iELISA has been validated mainly in other geographic settings [27] and its external validity for Somali cattle may vary due to local host and pathogen differences, the parallel use of a highly specific *msp1β* qPCR assay in this study strengthens confidence in our overall findings. Additionally, it should also be considered that MSP5 is highly conserved among *Anaplasma* species, and serological cross-reactivity with other species (e.g., *A. centrale* or *A. ovis*) cannot be excluded. As species-specific serological confirmation was not performed, this represents a limitation of the present study. Despite these limitations, this study represents the first molecular and serological investigation of bovine anaplasmosis in Somalia using a combination of diagnostic approaches. Our findings provide essential baseline data and underscore the need for broader, randomized epidemiological studies to more accurately assess the distribution and genetic variability of *A. marginale* in Somali cattle.

## Conclusions

Our results show high exposure and/or infection by *A. marginale* among cattle from the Lower Shabelle Region of Somalia. Ten novel Msp1a strains were identified, with SSR “81” being most common. Fifteen SSRs were characterized. Diversity analysis of 64 *msp4* sequences revealed 18 genotypes, clustering Somali sequences within one genotype, together with those previously detected in Russia and other African countries. Future studies should prioritize (i) probability-based sampling across multiple Somali regions, (ii) longitudinal herd-level studies to assess infection dynamics and endemic stability, and (iii) vector competence investigations to clarify the role of local tick species and mechanical transmission pathways. There is a need for further investigation of the occurrence of *Anaplasma* species other than *A. marginale* infecting cattle.

## Supplementary Information

Additional file 1.

## Data Availability

Data supporting the main conclusions of this study are included in the manuscript.
